# Acetylation of WCC is dispensable for the core circadian clock but differentially regulates acute light responses in *Neurospora*

**DOI:** 10.1016/j.jbc.2024.107508

**Published:** 2024-06-27

**Authors:** Bin Wang, Mark E. Adamo, Xiaoying Zhou, Ziyan Wang, Scott A. Gerber, Arminja N. Kettenbach, Jay C. Dunlap

**Affiliations:** 1 Department of Molecular and Systems Biology, Geisel School of Medicine at Dartmouth, Hanover, New Hampshire, USA; 2 Dartmouth Cancer Center, Geisel School of Medicine at Dartmouth, Lebanon, New Hampshire, USA; 3 Department of Biochemistry, Geisel School of Medicine at Dartmouth, Hanover, New Hampshire, USA

**Keywords:** WC-1, WC-2, acetylation, mono-methylation, period length

## Abstract

In the *Neurospora* circadian system, the White Collar Complex (WCC) formed by WC-1 and WC-2 drives expression of the *frequency* (*frq*) gene whose product FRQ feedbacks to inhibit transcriptional activity of WCC. Phosphorylation of WCC has been extensively studied, but the extent and significance of other post-translational modifications (PTM) have been poorly studied. To this end, we used mass-spectrometry to study alkylation sites on WCC, resulting in discovery of nine acetylation sites. Mutagenesis analysis showed most of the acetylation events individually do not play important roles in period determination. Moreover, mutating all the lysines falling in either half of WC-1 or all the lysine residues in WC-2 to arginines did not abolish circadian rhythms. In addition, we also found nine mono-methylation sites on WC-1, but like acetylation, individual ablation of most of the mono-methylation events did not result in a significant period change. Taken together, the data here suggest that acetylation or mono-methylation on WCC is not a determinant of the pace of the circadian feedback loop. The finding is consistent with a model in which repression of WCC’s circadian activity is mainly controlled by phosphorylation. Interestingly, light-induced expression of some light-responsive genes has been modulated in certain *wc-1* acetylation mutants, suggesting that WC-1 acetylation events differentially regulate light responses.

Circadian rhythms that have been found in most eukaryotes and certain prokaryotes orchestrate a wide variety of behavioral, metabolic, and molecular processes (1), allowing organisms to effectively utilize environmental resources. Dysfunction of the endogenous clock in humans has been implicated in many health issues and even severe disorders (2). Mechanistically, circadian clocks are composed of interlocked positive and negative element complexes: The latter progressively inhibit their own expression by deactivating the transcriptional activity of the former. *Neurospora crassa*, a genetically trackable model fungus sharing many basic cellular features with mammals, has been employed for circadian research for decades, facilitating great contributions to our understanding of the core clock operation (3, 4, 5). In *Neurospora*, the White Collar Complex (WCC) assembled from WC-1 and WC-2 acts as a signaling pivot for synchronizing the core oscillator that operates in darkness and can respond to environmental light signals (6). There exist two independent DNA elements in the promoter of the central circadian pacemaker gene *frequency* (*frq*): In constant darkness, WCC drives circadian expression of *frq* by binding to the *Clock box* (*C-box*) DNA element (7), while it associates with the *Proximal Light-Response Element* (*pLRE*) to activate *frq* transcription upon light exposure (6, 8).

FREQUENCY (FRQ), the gene product of *frq*, nucleates the formation of a multi-component complex, FFC (FRQ-FRH complex), with FRH (FRQ-interacting RNA helicase) (9, 10, 11) and CKI (Casein Kinase I) (12). In circadian cycles, FFC promotes phosphorylation of WCC at over 95 residues, most of which are involved only in the downregulation of circadian amplitudes (expression levels of circadian genes) but not in determining period length (13). However, circadian repression in the core oscillator arises from the phosphorylation of a small group of these residues (12, 13, 14, 15). FRQ itself also undergoes successive and extensive phosphorylations at over 110 residues (16, 17), many of which markedly impact the pace of the core circadian oscillator including prolonging or shortening the period length to variable extents as has been established by mutagenesis analyses of *frq* (18, 19, 20).

The mammalian functional orthologs of WC-1 and WC-2 are Bmal1 and its interacting partner, CLOCK (transcription factors bound to the *E-box* DNA element), which drive expression of circadian negative-element proteins Periods (Pers) and Cryptochromes (Crys), all of which feedback to repress the transcriptional activity of the Bmal1/CLOCK complex (reviewed in (21)). In mammals, multiple types of post-translational modifications (PTMs) have been identified on Bmal1 and CLOCK, including SUMOylation, ADP-ribosylation, O-GlcNAcylation, acetylation, ubiquitination, S-nitrosylation, and phosphorylation (22, 23, 24, 25, 26, 27). However, which PTM plays the major role in modulating the pace of the circadian oscillator remains unclear, probably partially due to gene redundancy of the circadian components. It was proposed that in the repression phase of the clock, CLOCK-mediated acetylation of Bmal1 at K537 in mice (equivalent to K538 of human Bmal1) is required for Cry-mediated repression of Bmal1/CLOCK (22). But this conclusion has been challenged by several lines of evidence: (1) Acetylation of Bmal1 occurs during the activation phase of a circadian cycle (28, 29); (2) Acetylation of K537 is mediated by Tip60 instead of CLOCK (23); (3) The K537R mutant of Bmal1 that abrogates K537 acetylation behaves like partial loss-of-function rather than being more active than WT (22) as would be expected for loss of a negative-acting PTM. In *Neurospora*, although phosphorylation is a determinant in depressing transcriptional activity of WC-1/WC-2 (see above), acetylation has also been detected in biochemical assays (30). Also, physical association between WC-1 and the histone acetyl-transferase NGF-1 has been reported as required for light responses (30), and has been associated with light-induction of gene expression. Despite these hints, however, the exact acetyl sites are wholly unknown, let alone their possible roles in the feedback mechanism dictating the core clock.

To explore possible alternative regulatory mechanisms in the circadian feedback loop, we studied WCC using mass-spectrometry to identify PTMs other than phosphorylation and found multiple acetylation and mono-methylation sites. Surprisingly, however, clock functions including period length and phase were little affected in most *wc-1* or *wc-2* mutants bearing single mutations at the acetylated or mono-methylated residues. Furthermore, even when all lysines on WC-1 or WC-2 were covered in the mutagenesis analysis, robust circadian oscillations were still observed. Altogether, the data here suggest that acetylation does not play an important role in modulating circadian periods of *Neurospora*, and acetylation of the circadian positive element proteins may be not a conserved mechanism for tuning their circadian activities.

## Results

### Identification of acetylated residues on WC-1 and WC-2

**Figure 1:**
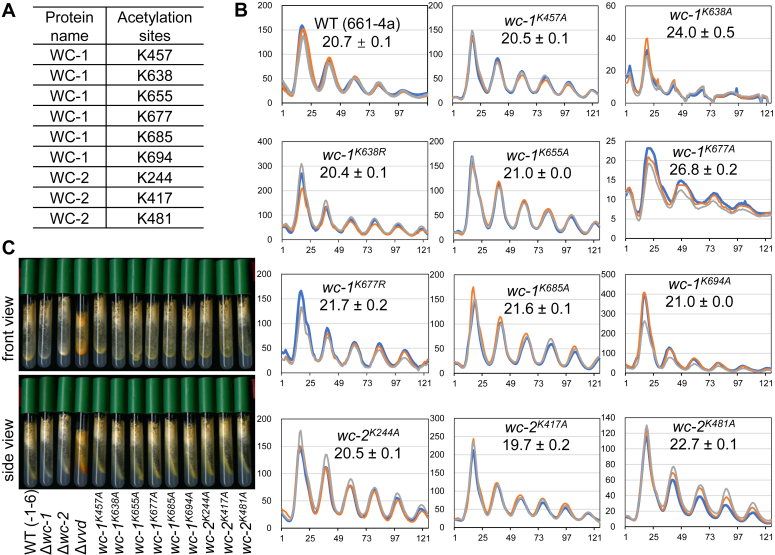
**Identification and mutagenesis study of acetylation on WC-1 and WC-2.***A*, list of identified acetylation sites on WC-1 and WC-2 by mass spectrometry. *B*, luciferase analysis of *wc-1* mutants bearing alanine mutations to the acetylation sites as stated. Strains were grown at 25 °C plus light overnight, and bioluminescence signals were recorded hourly with a CCD (charge-coupled device) camera after transfer of the strains to the dark also at 25 °C. Three replicates (in different colors) were plotted with the x-axis and y-axis meaning time (in hours) and arbitrary units of the bioluminescence-signal intensity, respectively. Period length was calculated from three replicates and reported as the average ± the standard error of the mean (SEM). All *wc-1* or *wc-2* mutants throughout this study were targeted to their native loci with a V5 at the C-terminus of WC-1 while WC-2 untagged. *C*, pigmentation and growth phenotypes of the *wc-1* and *wc-2* acetylation mutants. Strains were inoculated on Vogel’s minimal slants, cultured at 30 °C for 2 days, and then moved to 25 °C, LL for another day prior to being photographed. WT is “-1-6” in which WC-1 is V5-tagged at the C-terminus; Δ*vvd* serves as a control to show the overproduction of carotenoid due to highly active WCC in the light; Δ*wc-1* and Δ*wc-2* are negative controls.

In order to probe PTMs beyond phosphorylation on the WCC, WC-1 and WC-2 were purified from cultures grown in constant light (LL) and analyzed by mass spectrometry. In LL, FRQ is relatively highly expressed, leading to maximal repression of the circadian activity of WCC. Therefore, novel PTMs, if any, involved in WCC repression should be strongly enriched under LL just as FRQ-promoted phosphorylation events on WCC (13). Spectra were searched for evidence of acetylation, (mono-, di-, and tri-) methylation, and ubiquitination, all of which have been reported on Bmal1/CLOCK (see above), the mammalian functional orthologs of *Neurospora* WC-1/WC-2. Interestingly, six acetylation sites were found on WC-1 and three on WC-2 (Figs. 1*A*, S1, and Table S1) with a coverage of 99.1% for WC-1 and 97.7% for WC-2 (Fig. S2). This result is consistent with previous literature that used Western blotting to show that WC-1 is an acetylated protein (30). Mono-methylation was also detected on WCC (Table S1 and see below); however, we did not retrieve any ubiquitinated peptides from WC-1 or WC-2, probably in part because we did not attempt to inhibit proteasomes in the cells from which WCC was isolated. The data here indicate that the positive-element complexes in *Neurospora* and mammals (see above) are both alkylated at multiple residues.

### Mutagenesis analysis of acetylation sites on WCC

To examine the possible role(s) of WCC acetylation in the circadian clock, mutagenesis analysis (Fig. 1*B*) was performed to eliminate acetyl-sites found on WC-1 and WC-2 (Fig. 1*A*). Mutations in most of the WC-1 or WC-2 acetyl-sites, when changed individually (to alanine,) did not significantly impact period length (Fig. 1*B*) except for K677 on WC-1. *wc-1*^*K677A*^ showed a period of 26.8 h with low amplitude (compare y-axes), long period, and low amplitude being characteristic of *wc-1* or *wc-2* mutants with partially compromised transcriptional activity. For example, ER24, a *wc-2* mutant bearing a point mutation in the Zinc-finger DNA-binding domain rendering it partially defective in DNA binding, exhibits a markedly long period (28.6 h) (31); several *wc-1* mutants having partial deletions in the coactivator-recruitment domain show an approximately 25-h period (32). On the contrary, *wc-1* or *wc-2* mutants with enhanced circadian activity always display shortened periods with a high amplitude (13). K677 is situated in the linker region between PASB and PASC, both of which are required for WC-1 to be functional in driving *frq* expression and WC-2 interaction (33). Therefore, *wc-1*^*K677A*^ seems to be a partial loss-of-function mutant based on the long period as well as the low amplitude. Another piece of evidence comes from substitutions of arginine for lysine, a change that preserves the positive charge but prevents acetylation. *wc-1*^*580-1167K0R*^, in which all lysines (including K677) falling in aa 580 to 1167 of WC-1 were mutated to arginines, did not show a period-length adjustment (see below). *wc-1*^*K638A*^ displayed a 24-h period with a low amplitude (Fig. 1*B*). Due to the proximity of the mutation to PASB in *wc-1*^*K638A*^, the mechanism underlying period elongation of *wc-1*^*K638A*^ might be similar to that of *wc-1*^*K677A*^. Overall, the mutagenesis data suggests that most single acetylation events identified on WCC do not significantly impact the pace of the core oscillator.

Given that WCC is the principle blue light photoreceptor for the organism, and it directly controls the carotenoid biosynthetic pathway, light-induced pigmentation was examined for the acetylation mutants of *wc-1* and *wc-2*. These mutants in the light display an orange color on the minimal slants, which is similar to that of WT but quite distinct from the controls Δ*vvd* (deep orange), Δ*wc-1* (white), and Δ*wc-2* (white) (Fig. 1*C*), suggesting normal light-induced WCC dimerization (necessary for its activation) and normal WCC-VVD interaction (key for photoadaptation) in these mutants.

### Biochemical analysis of acetylation on WCC

**Figure 2:**
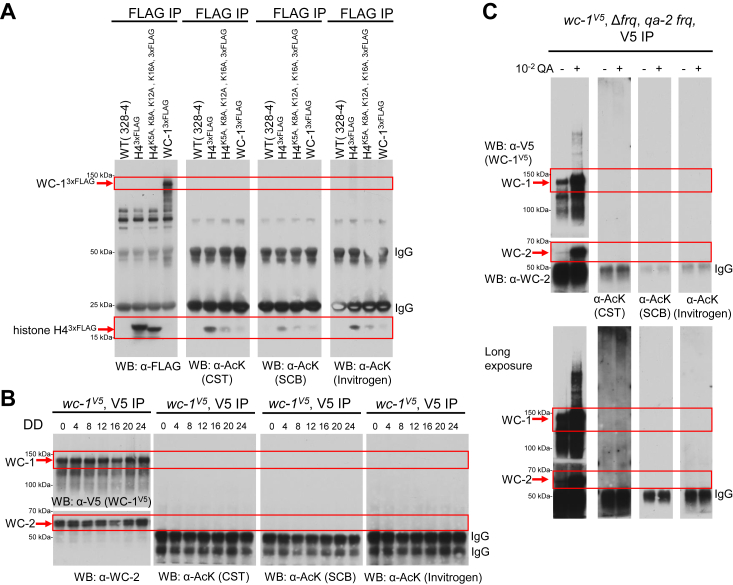
**Acetylation of WC-1 and WC-2 was not detected by Western blotting.***A*, quality control for acetyl-lysine antibodies. WT (328-4, untagged) served as a negative control for WB and IP, a positive control for acetyl-lysine-specific antibodies came from a *Neurospora* strain ectopically expressing a second copy of histone H4 with a C-terminal 3 x FLAG at the *csr-1* locus, and another negative control for acetylation is a strain that bore a second copy of histone H4 (also with a C-terminal 3 x FLAG tag at *csr-1*) carrying K5A, K8A, K12A, and K16A mutations, four conserved acetylation sites. WC-1 (tagged with 3 x FLAG at the C terminus) was isolated first by immunoprecipitation (IP) with FLAG resin. The three commercial acetyl-lysine antibodies were purchased from Cell Signaling Technology [CST], Santa Cruz Biotechnology [SCB], and Invitrogen and used in Western blotting each at a dilution of 1:1000. All *Neurospora* samples were cultured at 25 °C with light for approximately 24 h. *B*, acetylation of WC-1 and WC-2 was not detected by Western blotting from samples collected in a 24-h time course (with an interval of 4 h). V5-tagged WC-1 (along with WC-2) was isolated from the cell by V5 immunoprecipitation. Western blotting was performed with indicated antibodies. *C*, overexpression of FRQ does not induce WCC acetylation. In the strain of *wc-1*^*V5*^, Δ*frq*, *qa-2 frq*, FRQ was overexpressed by adding QA to the 0.1% glucose LCM medium at the final concentration of 10^−2^ M and immediately transferring the culture to the dark at 25 °C for 16 h. The same strain cultured under the identical condition but without QA throughout served as the negative control. WC-1 (V5 tagged) was isolated by immunoprecipitation with V5 resin and then Western blotting was carried out with indicated antibodies.

To follow the acetylation status of WC-1 and WC-2 more conveniently, we performed Western blotting with several commercially available antibodies. The three antibodies that we tried have been highly rated or cited by users for successfully detecting lysine acetylation on proteins from a broad range of organisms (see Experimental Procedures). To prevent false positive detection in Western blotting from nonspecific binding, WC-1 (tagged with 3 x FLAG at the C terminus) was first isolated from the lysate by immunoprecipitation (IP) prior to Western blotting with acetylated lysine-specific antibodies. The assay included three controls: WT (untagged) served as a negative control for Western blotting and IP, a positive control for acetyl-lysine-specific antibodies came from a *Neurospora* strain ectopically expressing a second copy of histone H4 with a C-terminal 3 x FLAG at the *csr-1* locus, and another strain as the negative control for acetylation bore a second copy of histone H4 (also with a C-terminal 3 x FLAG tag at *csr-1*) carrying K5A, K8A, K12A, and K16A mutations, four acetylation sites conserved widely among diverse organisms (34, 35). Following FLAG IP and Western blotting, specific bands with expected sizes corresponding to histone H4, histone H4^K5A, K8A, K12A, K16A^, and WC-1 appeared in samples from the transgenic strains, which were not seen in the untagged strain (Fig. 2*A*, the FLAG blot). Strong and specific histone H4 bands were observed with each of the three acetylated lysine (AcK)-specific antibodies (Fig. 2*A*, AcK antibodies from Cell Signaling Technology [CST], Santa Cruz Biotechnology [San Cruz], and Invitrogen). At the same position of histone H4, only very faint bands were seen from the sample of histone H4^K5A, K8A, K12A, K16A^ (denoted by the lower red box in Fig. 2*A*), probably due to weak non-specific reaction between the highly abundant unacetylated (K->A) histone H4 and the AcK antibodies. However, on any of the three Ack blots, no acetylation signal was detected at the position where WC-1 is supposed to appear (defined by the upper red box across the FLAG and three AcK blots in Fig. 2*A*). The strong and specific signals from the positive control (3 x FLAG-tagged histone H4) indicate that the three commercial antibodies against acetylated lysines worked nicely for proteins expressed and extracted from *Neurospora* but also suggests that acetylated WC-1 and WC-2 detected by mass spectrometry (Figs. 1*A* and S1) may only represent a small fraction of all the WC-1 and WC-2 molecules, consistent with the absence of circadian period changes in strains bearing single mutations to the acetylation sites of WC-1 or WC-2 (Fig. 1*B*). If control of WCC’s circadian activity involves acetylation, its level should oscillate in a circadian cycle, like what was seen for WCC phosphorylation (represented by WC-2 phosphorylation) (13, 14). To test this possibility, V5-tagged WC-1 was pulled down with V5 resin and then blotted with the three AcK antibodies that have been validated (Fig. 2*A*). The strong and specific WC-1^V5^ and WC-2 bands were noticed after IP enrichment but acetylation signals were not detected at the positions of WC-1 and WC-2 on any of the three Ack blots at any examined circadian time points even at DD24 when WCC is fully repressed (Fig. 2*B*). If acetylation of WCC is a driving mechanism in the circadian feedback loop, FRQ as the central negative-element protein for the core oscillator might control the acetylation of WCC as it does phosphorylation (14). To this end, FRQ was overexpressed by an inducible promoter, *qa-2*, in the dark for 16 h, and WC-1 (V5 tagged) along with WC-2 was isolated (by V5 IP) and blotted for the AcK antibodies. Relative to the no QA sample, the level of WC-1 and WC-2 was markedly and characteristically enhanced by quinic acid (QA)-induced FRQ overexpression, which is totally consistent with previous literature (13, 36) showing that FRQ promotes WCC phosphorylation, makes it leave the chromatin, and thereby protects it from being degraded in transcription, leading to its accumulation. Consistent with the data in Figure 2, *A* and *B*, WCC acetylation remains undetectable even when FRQ is overexpressed (Fig. 2*C*). Collectively, the biochemical data here suggest that acetylated WCC detected by mass spectrometry may only account for a very small fraction of the WCC population, and WCC activity seems unlikely to be determined by acetylation due to its low penetrance.

### Phenotypes of *wc-1* and *wc-2* mutants covering all lysine residues

**Figure 3:**
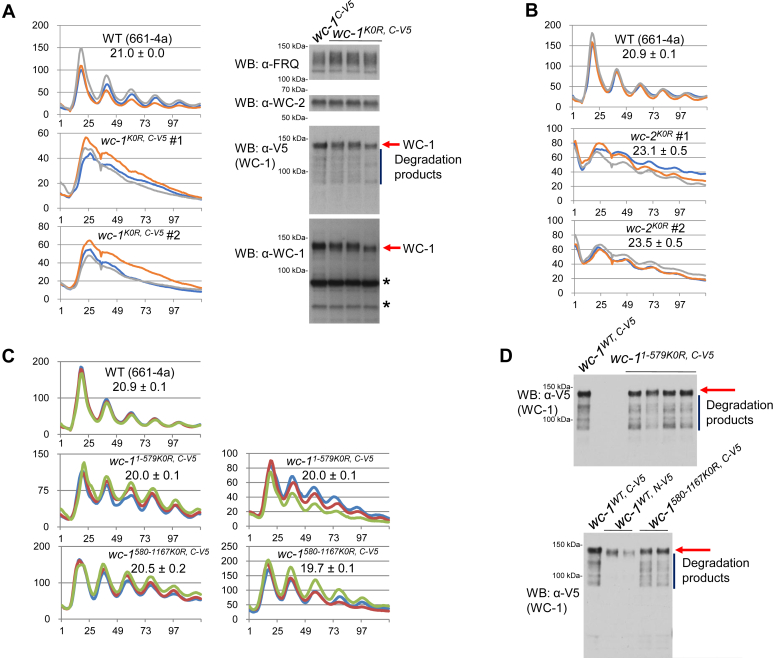
**Luciferase analysis of *wc-1***^***K0R***^**and *wc-2***^***K0R***^**.** “K0R” means that all lysine residues (in WC-1 or WC-2) were mutated to arginines. *A*, *left*, luciferase analysis of *wc-1*^*K0R*^; *right*, expression of FRQ and WC-1 in *wc-1*^*K0R*^. *B*, luciferase analysis of *wc-2*^*K0R*^. Clock synchronization was done by growing cultures at 25 °C with light overnight (∼16 h), and bioluminescence signals were recorded hourly with a CCD camera after moving the strains to darkness (at 25 °C). Three replicates were plotted with the x-axis and y-axis representing hours and arbitrary signal intensities, respectively. Period length was computed from the three replicates and presented as the average ± the standard error of the mean (SEM). WC-1 in *wc-1*^*K0R*^ bear a V5 tag at the C-terminus while WC-2 in *wc-2*^*K0R*^ is tagless. *wc-1*^*1-579K0R*^ and *wc-1*^*580-1167K0R*^ show a wild-type period length and normal WC-1 expression. *C*, luciferase analysis of *wc-1*^*1-579K0R*^ and *wc-1*^*580-1167K0R*^ at 25 °C. Synchronization, signal recording, and data plotting follow the same way as figures above. Three replicates (in three colors) were plotted with the x-axis and y-axis meaning time (in hours) and arbitrary light-signal intensities, respectively. *D*, expression of WC-1 in *wc-1*^*1-579K0R*^ (upper) and *wc-1*^*580-1167K0R*^ (*bottom*), both of which have a V5 at the C-terminus. *wc-1*^*V5*^, the control, also has a C-terminal V5 tag. Protein was extracted from cultures grown in the light at 25 °C. *Red arrows* point to the full-length WC-1 bands, and diffused bands (denoted with a vertical line) are degradation products of WC-1^V5^.

To test the combinatorial effects of acetylation events, if any, and also to study WC-1 acetylation events that might be missed in mass spectrometry, *wc-1*^*K0R*^ in which all lysines (a total of 49) of WC-1 were mutated to arginines was generated and assayed. *wc-1*^*K0R*^ displayed an arrhythmic clock in the luciferase assay which monitors the transcription activity of WCC at the *frq* promoter (Fig. 3*A* left), and levels of WC-1, WC-2, and FRQ in *wc-1*^*K0R*^ were all comparable to those in WT (Fig. 3*A* right). The arrhythmicity and reduced signal intensity (comparing y-axes) in *wc-1*^*K0R*^ are likely due to the large number of mutations (49 in total) that were introduced in the protein, reminiscent of what was seen in another mutant of *wc-1*, *wc-1*^*113A*^, in which all the 80 phosphosites along with 33 additional Ser or Thr were mutated together to alanines (13).

To examine the role of WC-2 acetylation in the core clock operation, we engineered *wc-2*^*K0R*^ in which all the lysine residues (25 in total) of WC-2 regardless of their acetylation status were mutated to arginines. *wc-2*^*K0R*^ displayed circadian rhythms for 4 days though its period length was lengthened slightly by ∼2 h (Fig. 3*B*) and the waveform of the rhythm was severely affected. While this likely reflects a side effect of a large number of mutations in the protein, the data indicate that WC-2 acetylation is not required for rhythmicity.

### Mutation of all lysines in either half of WC-1 did not alter the period length

To restore circadian oscillations in *wc-1*^*K0R*^ that were arrhythmic in the luciferase assay (Fig. 3*A*), two additional *wc-1* mutants derived from *wc-1*^*K0R*^ were engineered: *wc-1*^*1-579K0R*^ bearing all lysines from aa 1 to 579 (16 lysines) mutated to arginines while keeping aa 580 to 1167 unchanged and *wc-1*^*580-1167K0R*^ in which aa 1 to 579 remains WT but all lysines located in aa 580 to 1167 (33 lysines) were mutated to arginines. Both *wc-1* mutants, *wc-1*^*1-579K0R*^ and *wc-1*^*580-1167K0R*^, showed a robustly oscillating clock with a marginally shortened period length (Fig. 3*C*). The level of WC-1 in *wc-1*^*1-579K0R*^ and *wc-1*^*580-1167K0R*^ is almost identical to that in WT (Fig. 3*D*). Taken together, these data do not indicate an essential role for acetylation in modulating the circadian activity of WC-1 nor in controlling the operation of the core oscillator in *Neurospora*.

### Acetylation of WCC modulates expression of certain WCC target genes upon light exposure

**Figure 4:**
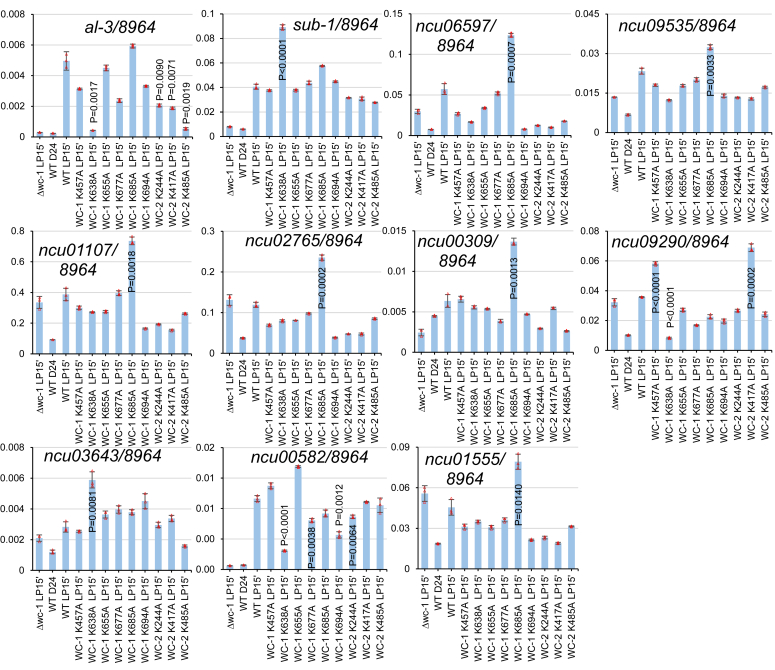
**Acute light responses in *wc-1* acetylation mutants.***Δwc-1* and WT serve as negative controls for the light response test. All the strains listed were cultured in the dark for 24 h and then given a 15-min light pulse except for WT that was only cultured in the dark for 24 h. RNA was extracted from the samples, reverse transcription (RT) was performed, and quantitative PCR (qPCR) was conducted with gene-specific primer sets. Columns with *p* values mean a reproducible change of expression in mutants *versus* WT. The data presented in the figure come from one experiment, while data obtained from the other independent biological repeat were deposited in Fig. S5. Two-tailed *p* values shown are less than 0.015 (https://www.graphpad.com/quickcalcs/ttest1/?format=SEM).

WCC is not only the central transcriptional activator for the clock but also directly and strongly induces transcription of light-responsive genes genome-wide upon a light pulse (37). To test whether the expression of light-inducible genes is affected by acetylation events on WC-1 or WC-2 (Fig. 1), the acetylation mutants of *wc-1* and *wc-2* were cultured in the dark for ∼24 h and thereafter exposed to strong light for 15 min. Reverse transcription (RT) followed by quantitative PCR (qPCR) was performed for mRNA extracted from the light-pulsed samples. The RT-qPCR data show that light induction of the *al-3* gene became undetectable in *wc-1*^*K638A*^, while that of *sub-1* increased prominently in the same mutant (Fig. 4). *al-3* expression was evidently repressed in *wc-2*^*K244A*^, *wc-2*^*K417A*^, and *wc-2*^*K485A*^. Light-activated expression of *ncu06597*, *ncu09635*, *ncu01107*, *ncu02765*, and *ncu00309* (37) was dramatically elevated in *wc-1*^*K685A*^ relative to that in WT (Fig. 4). Of these genes tested, only *sub-1*, *ncu06597*, *ncu09535*, and *ncu01107* were found to be direct targets of WCC (38), suggesting that the acetylation events on WCC might indirectly modulate the light signaling at the transcriptional level. These data here suggest that acetylation of primary photoreceptors, WC-1 and WC-2, differentially regulates acute light responses in the organism.

### Identification of mono-methylation sites on WC-1

**Figure 5:**
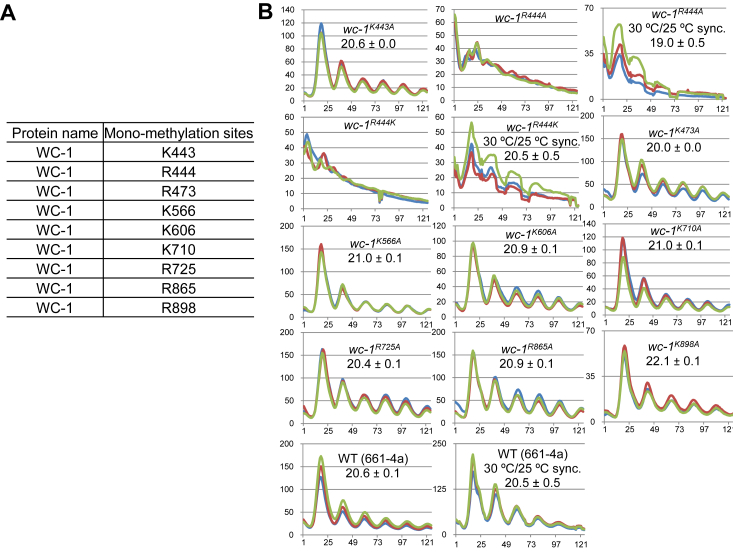
**Identification and mutagenesis studies of mono-methylation sites on WC-1.***A*, identification of mono-methylation sites on WC-1. *B*, luciferase analysis of *wc-1* mutants bearing alanine mutants to the mono-methylation sites as stated. Strains were grown at 25 °C or 30 °C as marked plus light overnight and transferred to the dark at 25 °C. Bioluminescence signals from the dark-grown strains were tracked hourly. Three replicates as shown in three colors are represented with time (in hours) as the x-axis and the signal intensity (arbitrary units) as the y-axis. The period length was calculated from the three replicates and displayed as the average ± the SEM. All *wc-1* mutants were appended by a V5 tag at the native locus.

From the mass-spectrometry data of WC-1 and WC-2, we also identified nine mono-methylation sites on WC-1 (Figs. 5*A* and S3) with a coverage of 51.4% for WC-1 and 46.7% for WC-2 (Fig. S4). We could not retrieve any di- or tri-methylated, or ubiquitinated peptides from WC-1 and WC-2, which might reflect the low abundance or absence of those modifications on WCC in the cell. The data here clearly indicate that WC-1 is also modified by at least two additional types of PTMs other than phosphorylation.

### Mutagenesis analysis of mono-methylation sites on WC-1

Like the minor effects of WCC acetylation on the clock (Fig. 1*B*), most mono-methylation sites (Fig. 5*A*) when individually mutated to alanine, did not cause evident period alterations, with the exception of R444 (Fig. 5*B*). Either R444A or R444K caused arrhythmicity, which might be due to the mutation at R444 interfering with the function of the LOV domain (aa 389–506) in light-medicated synchronization of the clock, similar to what was noticed in *wc-1*^*ΔLOV*^ (39). To synchronize the endogenous clock in a way independent of light, *wc-1*^*R444A*^ and *wc-1*^*R444K*^ mutants were grown at 30 °C (still in the light) for ∼24 h and then moved to the dark at 25 °C for recording WCC circadian activity *via* the *frq C-box*-driven luciferase. The luciferase data reveal that both mutants displayed a robust rhythm with a roughly WT period length (Fig. 5*B*). The data here are consistent with R444 mutations interfering with light signaling through the LOV domain but not impacting the clock, and suggest that like acetylation, mono-methylation on WCC is also not a determinant of the period length.

## Discussion

In this study, we identified acetylation and mono-methylation sites on WC-1 and WC-2, thereby extending beyond phosphorylation of the PTMs found on *Neurospora* clock proteins. Unexpectedly, mutating most of these modified residues individually did not lead to strong period changes, and biochemical analysis did not detect acetylation on WCC, suggesting that acetylation may be not highly penetrant in the WCC population. In the mammalian circadian system, multiple types of PTM events have been documented on the circadian positive element proteins Bmal1/CLOCK, which impact their circadian activity to variable extents (see above). CLOCK has been reported to possess acetyltransferase activity, and one of its substrates is also its interacting partner, Bmal1, whose acetylation at K537 facilitates Cryptochrome (Cry)-mediated repression of the Bmal1/CLOCK complex (22). However, there are contradictions regarding the timing and the biological relevance of K537 acetylation as well as the acetyltransferase in mediating the modification (see above). In contrast to acetylation on Bmal1/CLOCK, elimination of acetylation on WCC, however, did not result in a pronounced period alteration, suggesting that regulatory acetylation of circadian positive-element proteins is not a conserved mechanism across eukaryotic circadian systems. In the *Drosophila* clock, CYCLE (CYC), the functional ortholog of BMAL1, consists of essentially only the bHLH (basic Helix-Loop-Helix) and PASA/B (Per-Arnt-Sim A/B) domains without an extended C-terminal tail where K537 of Bmal1 is located, so CYC even lacks the acetylation site, K537, of BMAL1, which means that there is little evidence for conservation of regulation involving acetylation even across all animal clocks, much less from animals to fungi. Although multiple PTMs have been reported on Bmal1/CLOCK, which PTM plays a dominant role in dictating the clock operation remains unclear. In *Neurospora*, however, phosphorylation has been shown to be the major mechanism in tuning the circadian activity of WCC (12, 13, 14). Especially in a recent study (13), disruption of a small number among over 95 phosphosites that were mapped on WC-1 and WC-2 resulted in constitutively active WCC driving transcription of *frq* and *ccgs* (circadian-controlled genes), indicating that phosphorylation is the principal determinant for WCC repression.

**Figure 6:**
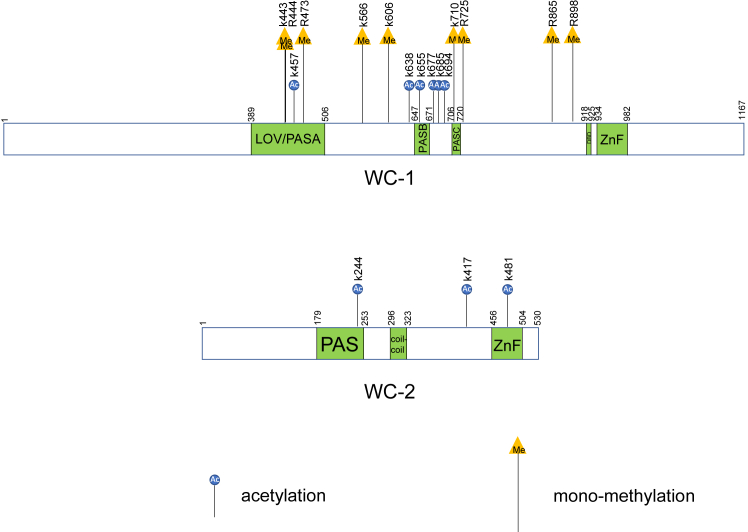
**Summary of acetylation and mono-methylation sites identified on WC-1 and WC-2.** A schematic diagram of WC-1 and WC-2 shows the positions of acetylated and mono-methylated residues identified in this study. WC-1 (1167 amino acids) contains LOV (light, oxygen, and voltage) (also known as PAS[Per-Arnt-Sim]A), two additional PAS (PASB and PASC), DBD (defective in DNA binding), and ZnF (zinc-finger) DNA-binding domains. WC-2 (530 amino acids) possesses PAS, coiled-coil, and ZnF domains. Blue circles that were labelled with “Ac” inside represent acetylation sites; orange triangles with “Me” inside mean mono-methylation residues. Above the *blue circles* and *orange triangles*, vertical letters and their following numbers annotate positions of the modified residues (either a lysine [K] or an arginine [R]).

WCC serves as the primary photoreceptor for mediating light-induced gene expression genome-wide and also for resetting the core clock through activating *frq* transcription *via* the *pLRE* element (see above). The RT-qPCR data (Fig. 4) reveal the roles of individual acetylation on WCC in regulating acute light responses. Elimination of acetylation at either K638 or K685 causes varying effects on the expression of different light-responsive genes, reflecting that acetylation at one individual residue may only modulate the expression of a subset of WCC-controlled genes. K638 and K685 are located near the PASB and PASC domains of WC-1 (Fig. 6) but are quite distant from the LOV domain, so the impact of the modification may be related to the overall structure of light-activated WCC instead of the formation of the WCC dimer.

## Experimental procedures

### *Neurospora* strains

*Neurospora* strain 661-4a (*ras-1*^*bd*^, *A*, *his-3*::*C-box*-driven *luciferase*), WT in luciferase assays, harbors the *C-box* DNA element from the *frq* promoter fused to the codon-optimized firefly *luciferase* gene (transcriptional fusion) at the *his-3* locus (40). *wc-1* and *wc-2* mutants were engineered using a published method relying on yeast homologous recombination-based integration of PCR fragments (32): Restriction-digested shuttle vectors *-1-6* (32) and *-2-1* (13) for *wc-1* and *wc-2* respectively were recombined with PCR (Thermo Fisher Scientific, Catalog # F549S) products amplified with primers bearing point mutations of *wc-1* or *wc-2*. Four primer pairs functioned as flanks in homologous recombination in a *Saccharomyces cerevisiae* strain FY834. All introduced point mutations at target sites were verified by Sanger sequencing in the Dartmouth Core facility with *wc-1*- or *wc-2*-specific primers. All these *wc-1* and *wc-2* mutants were targeted at the native loci. Plasmids bearing mutations were cut with *Ase*I (NEB, Catalog # R0526S) and *Ssp*I (NEB, Catalog # R0132S) and then purified with the QIAquick PCR Purification Kit (Qiagen, Catalog # 28104) for *Neurospora* transformation. *Neurospora* transformation *via* electroporation (settings: 1500 V, 600 Ω, and 25 μF) was performed using an electroporator (BTX, Model # ECM630) as previously described (20). The recipient strains for transforming the mutant constructs are Δ*wc-1*::*hph*; Δ*mus-52*::*hph*; *ras-1*^*bd*^ for *wc-1* and Δ*wc-2*::*hph*; Δ*mus-52*::*hph*; *ras-1*^*bd*^ for *wc-2* (13). All *wc-1* and *wc-2* mutants made in this research bear the *ras-1*^*bd*^ mutation and *frq C-box*-driven codon-optimized firefly *luciferase* gene at the *his-3* locus for circadian phenotype analyses, and all mutated WC-1 contain a V5 tag at the C termini for biochemical assays.

### Growth media

For vegetative growth, strains were cultured on complete-medium slants bearing 1 x Vogel’s, 1.6% glycerol, 0.025% casein hydrolysate, 0.5% yeast extract, 0.5% malt extract, and 1.5% agar (41). Sexual crosses between two *Neurospora* strains were conducted at 25 °C in the light on Westergaard’s agar plates containing 1 x Westergaard’s salts, 2% sucrose, 50 ng/ml biotin, and 1.5% agar (42). The Liquid Culture Medium (LCM) contains 1 x Vogel’s, 0.5% arginine, 50 ng/ml biotin, and 2% glucose.

### Protein extraction, immunoprecipitation, and Western blot

Protein extraction, IP, and subsequent WB were completed as previously documented (43, 44). LCM was used in culturing *Neurospora* for IP and WB; cultures were shaking at 125 rpm in the light at 25 °C overnight or as indicated. Vacuum-dried *Neurospora* tissue was frozen immediately in liquid nitrogen and ground to a fine powder with a ceramic mortar & pestle. 10 ml of protein-extraction buffer (50 mM HEPES [pH 7.4], 137 mM NaCl, 10% glycerol, 0.4% NP-40) was mixed with one tablet of cOmplete, Mini, EDTA-free Protease Inhibitor Cocktail (Roche, Catalog # 04693159001). The inhibitors-containing buffer was added to the ground powder followed by vortexing at top speed for 10 s and then resting on ice for another 10 s, which was repeated for a total of 2 min prior to incubation on ice for another 10 min. Thereafter, centrifugation (12,000-rpm centrifugation at 4 °C for 10 min) was done in order to remove insoluble debris.

For IP with *Neurospora* lysate, 2 mgs of extracts (precleaned by centrifugation at 12,000-rpm and 4 °C for 10 min) were incubated with 20 μl of FLAG resin (Sigma-Aldrich, Catalog # A2220) followed by rotating at 4 °C for 2 h. The protein-bound resin was washed (inverted 10 times) twice each with 1 mL of the protein-extraction buffer bearing protease inhibitors and afterward eluted with 100 μL of 5 × SDS sample buffer being heated at 99 °C for 5 min. 10 out of the 100-μL IP products were loaded per lane in a gel.

For WB, 15 μgs of centrifugation (12,000 rpm at 4 °C for 10 min)-cleared whole cell lysate were loaded per lane in a commercial 3 to 8% 1.5-mm x 15-well Tris-Acetate SDS gel (Thermo Fisher Scientific, Catalog # EA03785BOX), electrophoresing with 1 x NuPAGE Tris-Acetate SDS Running Buffer (Thermo Fisher Scientific, Catalog # LA0041). Applications of custom rabbit FRQ, FRH, WC-1, and WC-2 antibodies in WB have been reported previously (39). Antibodies against acetylated lysines include Acetylated-Lysine Mouse mAb (Ac-K-103) (Cell Signaling Technology [CST], Catalog # 9681), Acetylated Lysine Monoclonal Antibody (1C6) (Invitrogen, Catalog # MA1-2021), and Ac-lysine Antibody (AKL5C1) (Santa Cruz Biotechnology, Catalog # sc-32268), each of which was employed in Western blotting at a dilution of 1:1000.

### Identification of acetylation and mono-methylation sites on WC-1 and WC-2 by mass spectrometry

Purified WC-1 and WC-2 were processed and analyzed by LC-MS/MS (liquid chromatography-coupled tandem mass spectrometry) as previously described (13, 32). Raw data were searched against a custom database containing WC-1 and WC-2 sequences with a peptide mass tolerance of 1 Da, static modification carbamidomethyl (C) +57.02146, and dynamic modifications oxidation (M) +15.99491 and acetylation (K) +42.010565 or monomethylation (K, R) +14.01565.

### Luciferase-reporter assays

Luciferase assays were done following former methods (45, 46). 96-black well plates (0.8 ml of the luciferase-assay medium in each well) were inoculated with conidial suspension (conidia in water), and the inoculants were grown at 25 °C in the presence of light for 16 to 24 h and then transferred to the dark at 25 °C for monitoring light production. Medium used for luciferase assays bore 1 x Vogel’s salts, 0.17% arginine, 1.5% Bacto Agar, 50 ng/ml biotin, 0.1% glucose, and 12.5 μM luciferin (GoldBio, Catalog # LUCK-2G). Bioluminescence signals were tracked with a CCD (charged-coupled device) camera every hour, raw data were acquired with ImageJ and a custom macro, and period lengths were manually determined. Raw data from three or four replicates were shown in the figures, and time (in hours) on the x-axis was plotted against the signal intensity (arbitrary units) on the y-axis. WT used in luciferase assays was 661-4a (*ras-1*^*bd*^, *A*) with the codon-optimized firefly *luciferase* gene driven by the *frq C-box* at the *his-3* locus.

### RT-quantitative PCR

TRIzol reagent (Life Technologies) and SuperScript III first-strand synthesis kit (Life Technologies) were used to isolate *Neurospora* mRNA and to synthesize cDNA (from 3 μg of mRNA) respectively (37). Real-time PCR was conducted with iTaq Universal SYBR green Supermix (BIO-RAD, Catalog # 1725121) using an ABI 7500 Fast system. Sequences of primers against WCC target genes were obtained from prior publications (37, 45, 46). *ncu08964* served as an internal normalizer for RT-qPCR, which is constantly expressed under different nutrient or circadian conditions.

## Data availability

Mutants made in this study are available upon request. All data that were used to draw conclusions in the article have been provided in the figures.

## Supporting information

This article contains supporting information (47).

## Conflict of interest

The authors declare that they have no conflicts of interest with the contents of this article.
