# Supplementary material for: Acetylation of WCC is dispensable for the core circadian clock but differentially regulates acute light responses in Neurospora
Source: J Biol Chem. 2024 Jun 27;300(8):107508. doi: 10.1016/j.jbc.2024.107508 (PMC11325773; doi:10.1016/j.jbc.2024.107508)
Supplement: Supporting Information [file mmc3.docx]

**Supporting Table S1** List of identified acetylated and mono-methylated peptides derived from WC-1 and WC-2 by mass spectrometry. In the table, “*”, “~”, and “!” indicate oxidation, acetylation, and mono-methylation, respectively.

**Supporting Figure 1** Mass spectrometry spectra of acetylated peptides from WC-1 and WC-2. WC-1 and WC-2 were purified from cultures that grew in the light at 25 °C. Digestion of WC-1 and WC-2 was carried out with either proteinase K or trypsin as indicated in the Supporting Table 1. The spectra of the acetylated peptides were generated using IPSA (Interactive Peptide Spectra Annotator) (47). The x-axes in the charts are the ratio of mass to charge (m / z), while the y-axes are relative signal intensities (labelled as “relative abundance [%]”). Red arrowheads point to residues that are acetylated. In the annotated spectra, lower case “c” means carbamidomethylcysteine; lower case “m” represents oxidated methionine; lower case “k” indicates acetylated lysine. Nine acetylated peptide species of WC-1 and WC-2 were discovered in the analysis, and all the acetylated peptides of WC-1 and WC-2 have been deposited in the Supporting Table 1.

**Supporting Figure 2** Coverage maps of WC-1 and WC-2 in acetylation analysis. Residues covered by mass spectrometry are in red, while undetected residues are in grey.

**Supporting Figure 3** Mass spectrometry spectra of mono-methylated and unmethylated peptides from WC-1. WC-1 and WC-2 were isolated from light-grown cultures (at 25 °C), and digested proteinase K or trypsin as listed in the Supporting Table 1. The spectra of the mono-methylated peptides were produced by IPSA (Interactive Peptide Spectra Annotator) (47). In the spectra, lower case “c” denotes carbamidomethylcysteine; lower case “m” means oxidated methionine; lower case “r” or “k” represents mono-methylated arginine or lysine. The x-axes in the charts represent the mass to charge ratio (m / z), while the y-axes mean relative abundance [%]. Ten mono-methylated peptide species of WC-1 were found in the analysis, and all the mono-methylated WC-1 peptides have been put in the Supporting Table 1. Red arrowheads indicate WC-1 residues that were found to be modified by mono-methylation. Two types of mono-methylated peptides have been identified at the residue K443 of WC-1.

**Supporting Figure 4** Coverage maps of WC-1 and WC-2 in mono-methylation analysis. Mass spectrometry-identified residues were labeled in red and the rest are in grey.

**Supporting Figure 5** RT-qPCR data obtained from another independent biological repeat (relative to Fig. 4) using the *wc-1* and *wc-2* acetylation mutants (Fig. 1B) bearing alanine mutations to individual acetylation sites as indicated. P values shown are two-tailed and less than 0.015 (https://www.graphpad.com/quickcalcs/ttest1/?format=SEM).
